# HARNet in deep learning approach—a systematic survey

**DOI:** 10.1038/s41598-024-58074-y

**Published:** 2024-04-10

**Authors:** Neelam Sanjeev Kumar, G. Deepika, V. Goutham, B. Buvaneswari, R. Vijaya Kumar Reddy, Sanjeevkumar Angadi, C. Dhanamjayulu, Ravikumar Chinthaginjala, Faruq Mohammad, Baseem Khan

**Affiliations:** 1 Department of Computer Science and Engineering, SRM Institute of Science and Technology, Vadapalani, Chennai, Tamil Nadu 600026 India; 2 Department of Electronics and Communication Engineering, St. Peter’s Engineering College, Dhulapally, Hyderabad, 500100 India; 3 Department of Computer Science and Engineering, St Mary’s Group of Institutions, Hyderabad, 500100 India; 4 Department of Information Technology, Panimalar Engineering College, Poonamallee, Chennai, Tamil Nadu 600123 India; 5 Department of Computer Science and Engineering, Koneru Lakshmaiah Education Foundation, Vaddeswaram, Andhra Pradesh 522502 India; 6 Department of Computer Science and Engineering, Nutan College of Engineering and Research, Talegaon Dabhade, Pune, 410507 India; 7 School of Electrical Engineering, Vellore Institute of Technology, Vellore, India; 8 School of Electronics Engineering, Vellore Institute of Technology, Vellore, Tamil Nadu India; 9 Department of Chemistry, College of Science, King Saud University, P.O. Box 2455, 11451 Riyadh, Kingdom of Saudi Arabia; 10 Department of Electrical and Computer Engineering, Hawassa University, Hawassa 05, Ethiopia

**Keywords:** Human action recognition (HAR), Deep learning, CNN, Feature-based approaches, Accuracy, Energy science and technology, Engineering

## Abstract

A comprehensive examination of human action recognition (HAR) methodologies situated at the convergence of deep learning and computer vision is the subject of this article. We examine the progression from handcrafted feature-based approaches to end-to-end learning, with a particular focus on the significance of large-scale datasets. By classifying research paradigms, such as temporal modelling and spatial features, our proposed taxonomy illuminates the merits and drawbacks of each. We specifically present HARNet, an architecture for Multi-Model Deep Learning that integrates recurrent and convolutional neural networks while utilizing attention mechanisms to improve accuracy and robustness. The VideoMAE v2 method (https://github.com/OpenGVLab/VideoMAEv2) has been utilized as a case study to illustrate practical implementations and obstacles. For researchers and practitioners interested in gaining a comprehensive understanding of the most recent advancements in HAR as they relate to computer vision and deep learning, this survey is an invaluable resource.

## Introduction

**Figure 1:**
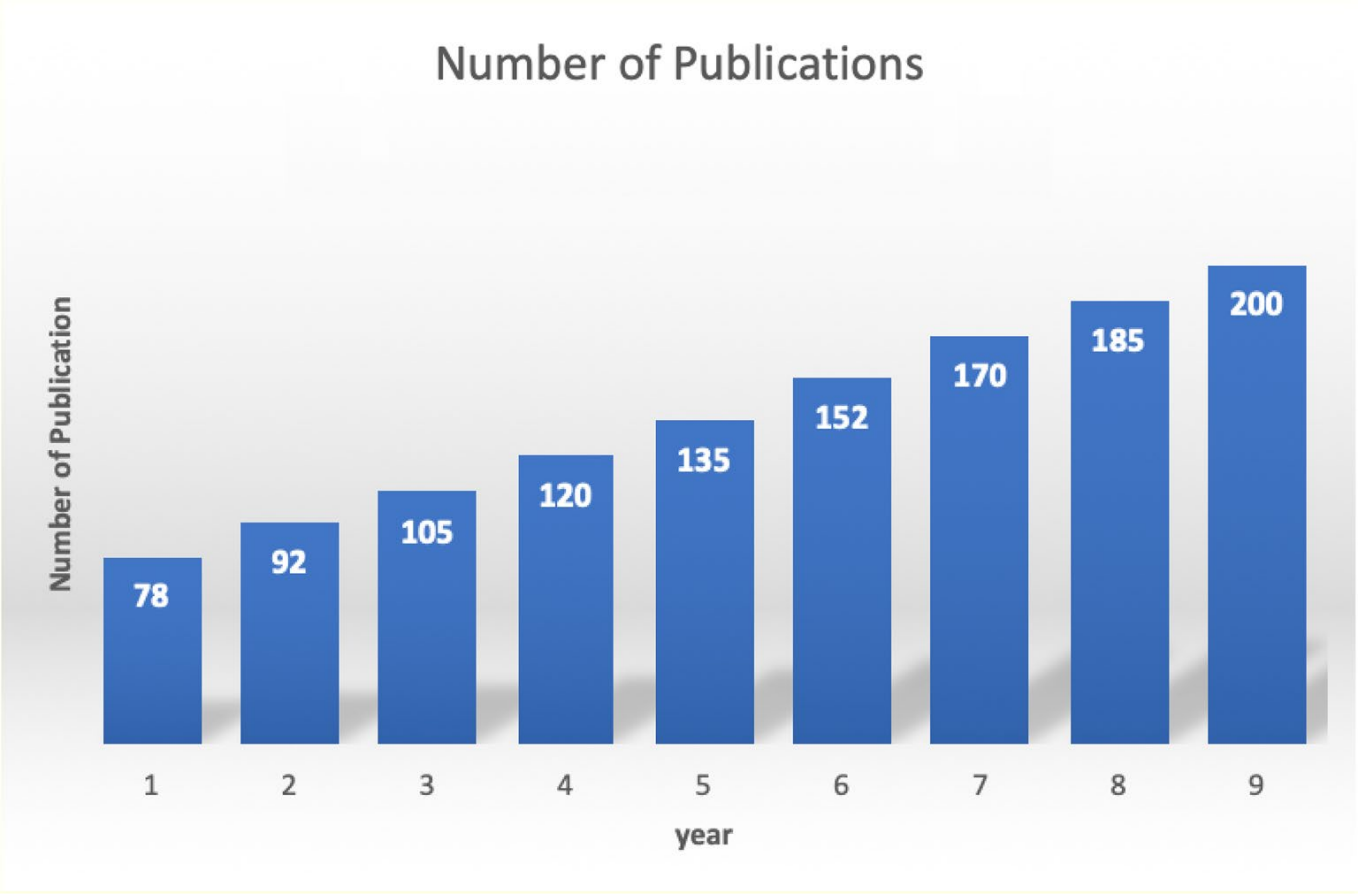
HAR related Publication in last few years.

Human activity recognition (HAR) is an area that is getting more and more attention from researchers. This rise in attention is due to HAR’s important role in many different applications. As technology gets better, it becomes more important to understand how people act in complex ways. HAR is at the front of the pack and promises to have huge effects on healthcare, security, and engaging technologies^1^. Researchers are becoming more and more interested in HAR, which shows how it could change smart environments, personalized healthcare, and how people connect with computers. In this changing world, studying HAR not only helps solve current problems, but it also opens the door to huge steps forward in understanding and using the complexities of human behavior^2^. Figure 1 shows the trend in research publications related to human action recognition over the past few years by providing valuable insights into the dynamic nature of human action recognition research over time.

Human activity recognition (HAR) is useful in many fields because it is flexible and has a big effect. Figuring out what people are doing and why they are doing it has become a key skill for making progress and finding answers in many areas. Take a look at how HAR covers a wide range of areas: But knowing what people do is still a difficult task that comes with its own problems. Accurate recognition is hard because people’s movements, the surroundings, and the details of the situation can change quickly^3^. Using cutting-edge tools and methods, researchers are working hard to solve these problems.

1. HAR is an important part of smart surveillance systems because it helps computers find strange or suspicious actions in public places. Real-time monitoring is possible with this technology, which makes crowded areas, transport hubs, and key infrastructure safer.
2. In healthcare, wearable gadgets that can do HAR can keep an eye on patients and give important information about their daily lives. HAR helps find falls in older people, spot early signs of neurodegenerative diseases, and keep people doing their rehabilitation routines.
3. HAR improves interactive games by adding moves from the real world to the games. Virtual characters can copy what users do, which makes the entertainment experience more realistic and interesting.
4. HAR is used in schools to see how engaged students are and to make sure that they are getting the most out of their learning. HAR is used in virtual reality (VR) and augmented reality (AR) apps to create realistic and flexible training environments.
5. By looking at how people and cars move in smart cities, HAR technologies help make traffic run better.

**Figure 2:**
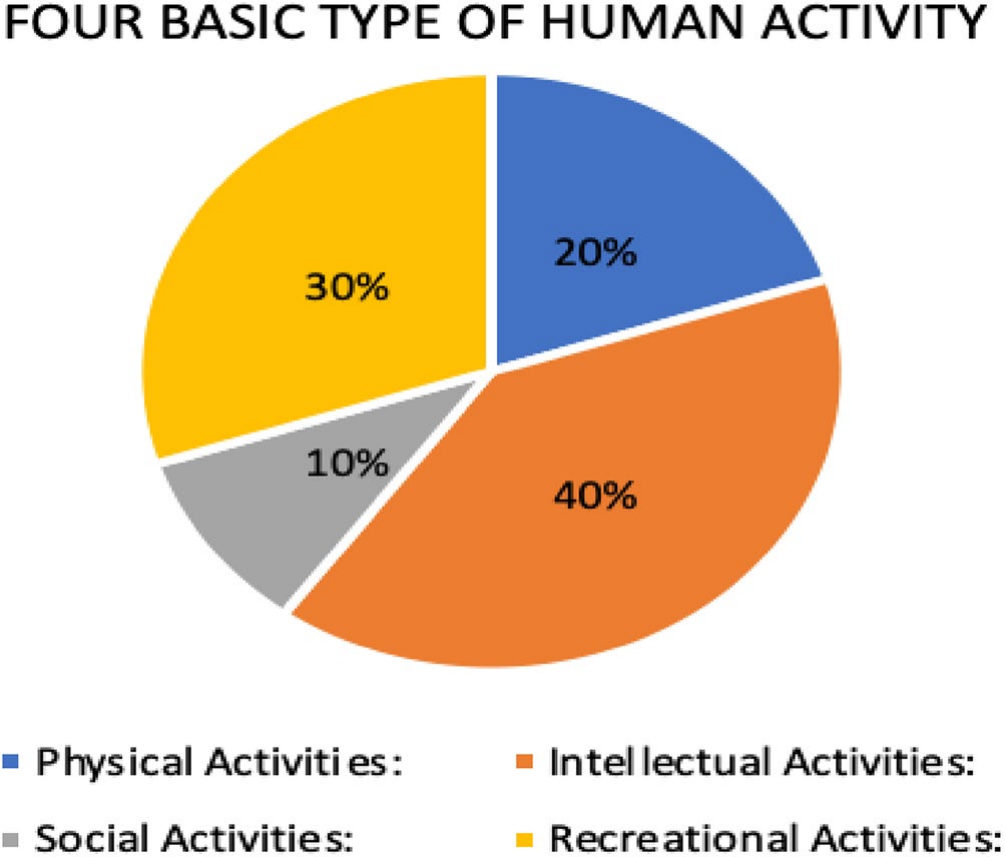
Categorization of human activities into four fundamental types.

Convolutional Neural Networks (CNNs) have become one of the most important new technologies in HAR. This introduction sets the stage for looking at the history of HAR, focusing on its practical importance, recent progress, and the key role that CNNs have played in helping us learn more about how people behave. The broad division of human activities into four basic categories—physical, intellectual, social, and recreational—is depicted in this schematic diagram^4^. The Fig. 2 shows a framework for visualizing the wide variety of behaviors that define human contact and engagement. A range of activities are included in each area, which reflects the complexity of human experience and behavior.

### Our contributions

1. An in-depth look into human activity identification using computer vision.
2. A thorough examination of traditional and deep learning-based action recognition systems.
3. The development of a versatile framework for recognizing human actions in videos.
4. A fresh taxonomy is proposed to classify various techniques in the sector.
5. A detailed description of recent work that is related to the proposed taxonomy.
6. Exploration of issues and identification of emerging trends.
7. Techniques, frameworks, research methodologies, datasets, difficulties, and future prospects are all organized in a systematic manner.
8. A succinct conclusion detailing potential future research directions.

Our paper was organized as follows, Overview of human action recognition techniques in “Overview of human actions” section. A generalized framework for the identification of human actions is presented in “Human action recognition framework” section. Introduction to human action recognition research methods and taxonomy, with reviews of feature extraction and activity categories, in “Human action recognition research methods and taxonomy” section. This section also encompasses evaluations of machine learning and handcrafted techniques, such as deep learning, in terms of their performance on a variety of datasets.

Exploration of well-known public datasets and methods for human action recognition in “Exploration of well-known public datasets and methods for human action recognition” section. A discussion of evaluation metrics and performance across various datasets is presented in “Evaluation of metrics and performance” section. An analysis of the challenges, prospects, and forthcoming trends in human activity recognition constitutes in “Challenges, prospects, and forthcoming trends in human activity recognition” section. In “Conclusion” section comprises the conclusion and a discussion of possible directions for future research.

## Overview of human actions

This study explores the broad field of human activity recognition by concentrating on techniques that have developed over time, with a particular focus on feature extraction and the kinds of activities examined^5^. The field of human activity detection research has seen a clear division of approaches based on feature extraction and the type of activities being studied. Significant advancements have been achieved in machine learning research, which now divide human action recognition techniques into three main groups: fully-automated deep learning-based approaches, machine learning techniques, and manually-built features.

### Hand-built features versus machine learning versus deep learning

There are several different ways used in human action detection, including fully-automated deep learning-driven methods, machine learning techniques, and manually-built features. Limitations include human identification restrictions, inaccurate posture estimates, camera motion, occlusion, and difficulties in complicated circumstances are frequently encountered by recognition algorithms that rely on manually-built features^6^. However, when it comes to extracting features from photographs, machine learning techniques—such as deep learning—perform better than handcrafted features.

### Depth sensors and Azure Kinect

The incorporation of depth sensors has greatly improved human posture estimation, yielding precise and real-time data regardless of changes in the foreground or backdrop. Systems that use skeletal sequences and depth data to recognize human actions have shown good accuracy while requiring little processing power. The debate encompasses a range of depth camera types, such as time of flight (TOF), triangulating, and structured light (SLT) cameras. An examination of Microsoft’s Azure Kinect sensor highlights its sophisticated capabilities, which include a microphone array, RGB and depth cameras, and support innovation in AI and Internet of Things applications.

### Deep learning strategies

It has been demonstrated that automated feature learning using deep learning techniques is superior than manually-built features. An overview of efforts to use deep learning techniques for feature extraction from RGB, skeletal, and depth data is provided in this work. The data provide a multimodal approach to feature learning, encompassing optical flow information, depth information, skeletal sequences, and overall outlook features^7^. The investigation encompasses optical flow data, skeletal and depth data, and visual patterns, demonstrating the variety of inputs available to deep learning networks. The importance of action feature extraction has recently increased, mostly due to deep learning-based high-efficiency posture estimation methods.

The two different facets of human action recognition that the research distinguishes between are action categorization and detection. The process of classifying activities in divided films into basic and complex categories, as well as figuring out their start and end times and spatial locations, is known as action categorization. On the other hand, related study fields like object recognition, deep learning, and human posture estimation have made human action detection more well-known.

Four levels can be used to classify the complexity of human actions: atomic, individual, human-to-object, and group actions. Atomic actions are the fundamental motions of the parts of the human body, whereas individual actions are those carried out by a single person, such walking or punching. Group activities and person-to-object interactions both entail interacting with things or several people, demonstrating the variety of human endeavors.

To sum up, this study offers a thorough overview of human activity detection approaches, ranging from conventional manually-built features to the cutting-edge capabilities of deep learning. It makes its way through the subtleties of depth sensors, the revolutionary effects of Azure Kinect, and the changing field of deep learning techniques. A more sophisticated knowledge of the complex field of human activity recognition is made possible by the distinctions between action categorization and detection, as well as the complex classification of human actions.

The field of human action recognition is actively being advanced by numerous organizations and research groups. Here are a few noteworthy ones:

#### FAIR, or Facebook AI research

FAIR is renowned for its noteworthy advancements in deep learning and computer vision. Two-stream network development is one of the many facets of human action recognition that FAIR researchers have worked on.

#### Google research

The vanguard of developments in computer vision and machine learning has been achieved by Google Research. Their contributions include the use of large-scale datasets for action recognition and research on deep learning systems.

#### Microsoft research

Microsoft Research has carried out a great deal of computer vision research, especially in the fields of depth sensing, action identification, and the creation of sensors such as Azure Kinect.

### (SAIL) Stanford artificial intelligence lab

SAIL is a prominent artificial intelligence research group at Stanford University. With applications in human action recognition, their work combines robotics, machine learning, and computer vision.

#### Oxford University’s visual geometry group (VGG)

The University of Oxford’s VGG is well known for its computer vision research. Among their contributions to action recognition has been the creation of benchmark datasets by VGG researchers.

#### Massachusetts Institute of Technology: Computer Science and Artificial Intelligence Lab (MIT CSAIL)

MIT CSAIL carries out innovative research in a range of AI domains. In order to better understand action recognition, they are investigating new algorithms and structures.

#### (BAIR) Berkeley Artificial Intelligence Research Lab at the University of California, Berkeley

BAIR at UC Berkeley conducts robotics, computer vision, and machine learning research. Their research on deep learning architectures has consequences for the identification of human actions.

#### Adobe study

Computer vision is one of the many research topics that Adobe Research works on. They have made significant advances to action recognition and video analysis.

#### NVIDIA study

In the development of GPUs and AI technologies, NVIDIA is a major participant. Action recognition has been studied by NVIDIA Research, particularly in relation to deep learning and.

#### Intelligent Sensory Information Systems (ISIS)—University of Amsterdam

Sensory information systems are the main focus of ISIS at the University of Amsterdam. They have conducted studies on the identification of human actions using multimodal data.

#### Max Planck Institute for Informatics

This German institute is well-known for its computer science research. Their contributions to the realm of action recognition and computer vision have been noteworthy.

## Human action recognition framework

**Figure 3:**
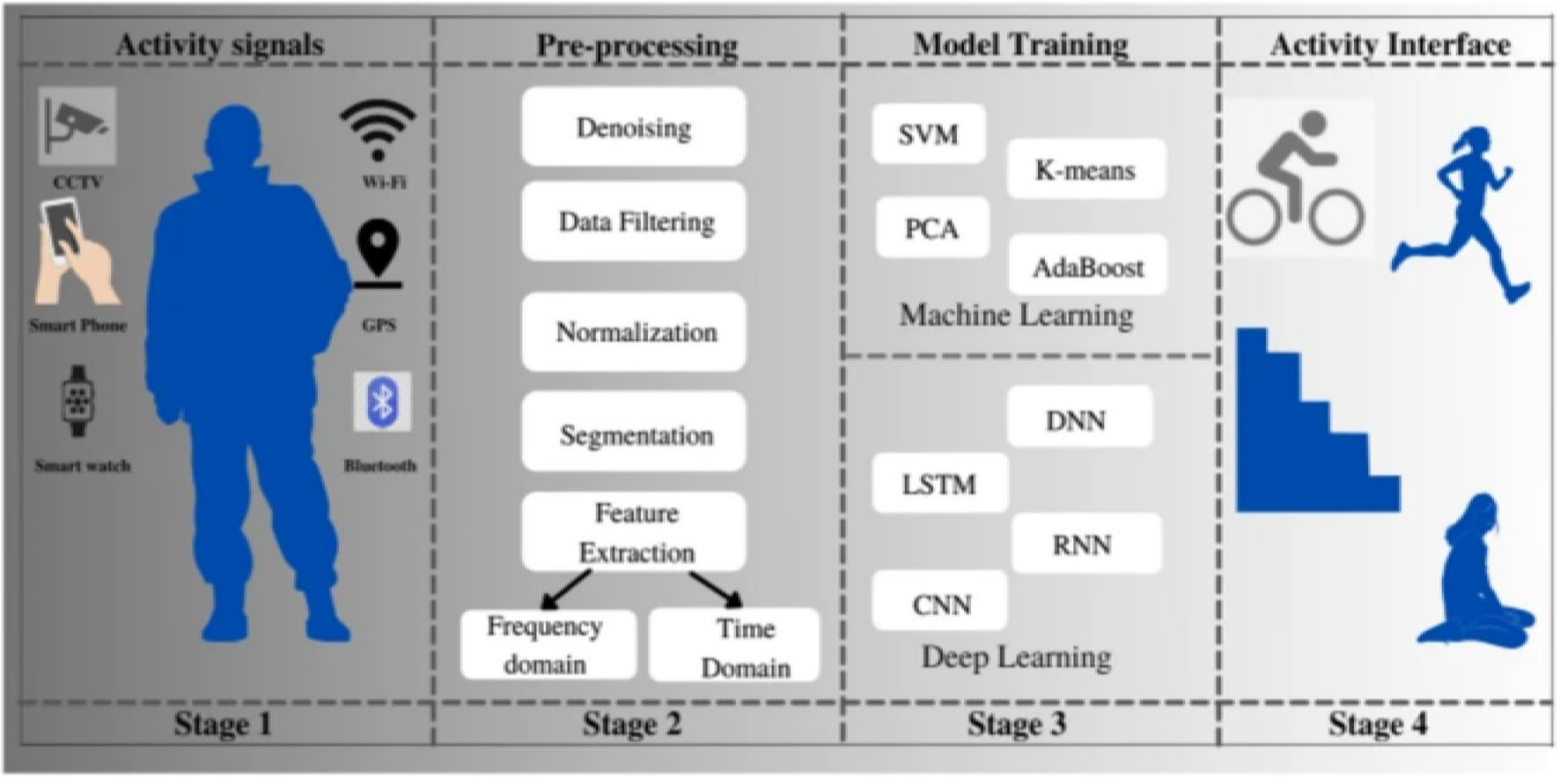
HAR framework^5^.

Early investigations in the field of Human Activity Recognition (HAR) revealed two basic methodologies based on the type of data analyzed: vision-based and sensor-based. The study of photographs or videos acquired by optical sensors, such as CCTV cameras, is used in vision-based HAR. Video-based systems for recognizing gestures and activities have received a lot of attention, especially in security, surveillance, and interactive applications. Sensor-based^8^. HAR, on the other hand, investigates raw data from wearable sensing devices, with wearable gadgets providing as examples^9^. Sensors’ efficiency is dependent on proximity and the sensor’s capacity to recognize specific behaviors. The HAR framework is made up of four major components: data collection (capturing data via optical sensing), pre-processing (improving collected data via normalization and resizing), learning or training (extracting features via machine learning and deep learning), and activity recognition or classification (using learned features to identify specific human actions). Figure 3 shows the HAR Framework. This paradigm offers a systematic way to comprehending and recognizing human actions, emphasizing the breadth and usefulness of HAR across multiple domains.

## Human action recognition research methods and taxonomy

The present work focuses on action classification, which encompasses a wide range of human actions categorized into four semantic levels: atomic, behavior, interaction, and group. The classification system explores the complexities of individual movements, overarching behaviors, interpersonal relationships, and aggregate group activities.

**Figure 4:**
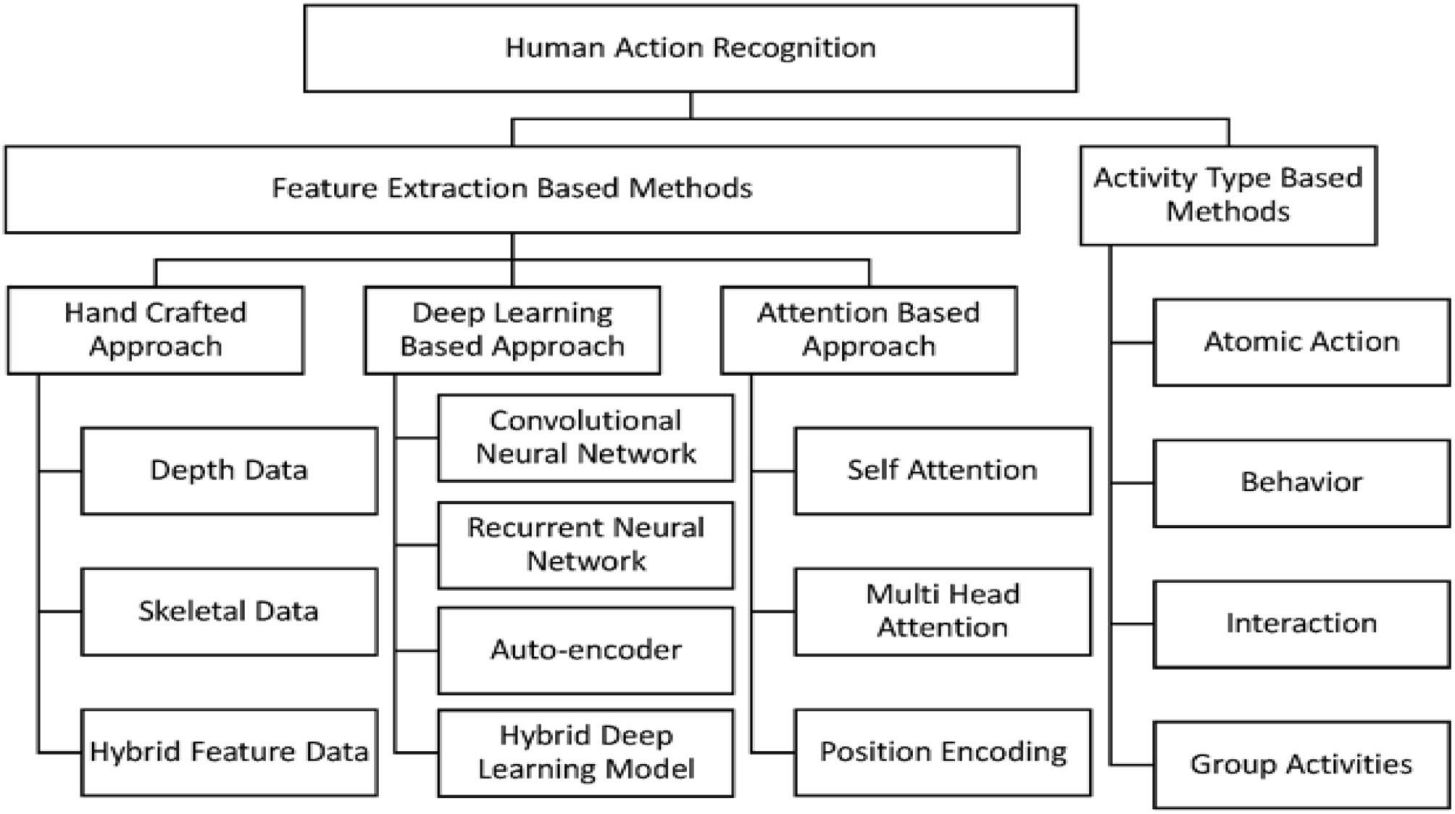
HAR research methods^31^.

The study tries to comprehensively analyze human behaviors by examining several semantic levels, ranging from basic movements to intricate group dynamics, in order to gain a nuanced knowledge of their richness and complexity. This comprehensive method guarantees a thorough examination of the varied aspects involved in recognizing human activity, offering significant insights into the complexities of actions across different semantic levels^10^. Figure 4 shows the Research and Taxonomy methods involved in the HARNET.

### Representation method in feature extraction-based action recognition

The process of feature extraction is very important in the field of Human Action Recognition (HAR) for figuring out and grouping different human actions^11^. This method involves turning raw data, which is usually video clips or basic data, into features that show what actions are really about. There are two main ways to describe things:

1. Representations of spatial and temporal elements:

This method is based on incorporating both spatial and temporal elements into action patterns^12^. It involves encoding how things or body parts are arranged in space and keeping track of how they change over time, giving a complete picture of activities.

Tips and tricks: Some common ways to show space and time are space–time volumes, 3D histograms of gradient orientations (HOG3D), and trajectory-based descriptions.

2. Representations based on skeletons:

This method uses skeletal data to show how people move and what they do by looking at how important body parts are arranged and how they move. It gives a short picture of pose and movement, which makes action recognition work well. To get a Well-focused picture, features like joint coordinates, joint angles, or relative distances between joints must be extracted from skeletal data.

3. Approaches to Feature Extraction Based on Depth:

Depth Maps: Methods that use depth use data from tools that measure how far away things or body parts are. It is possible to get features like depth value histograms or depth motion history pictures from depth maps, which show how space is organized^13^. Features based on skeletons for depth: Using spinal data from depth sensors, this method improves how actions are shown by pulling out details like joint angles, speeds, or accelerations in three dimensions. Feature extraction is one of the most important tools for figuring out how people act^14^. It lets recognition models find trends and make smart classifications by picking out and showing the most important traits. Whether using depth-based or space–time representations, feature extraction helps us understand human actions in more detail. This has led to progress in Human Action Recognition in many areas, such as robotics, human–computer interaction, and surveillance^15^.

**Figure 5:**
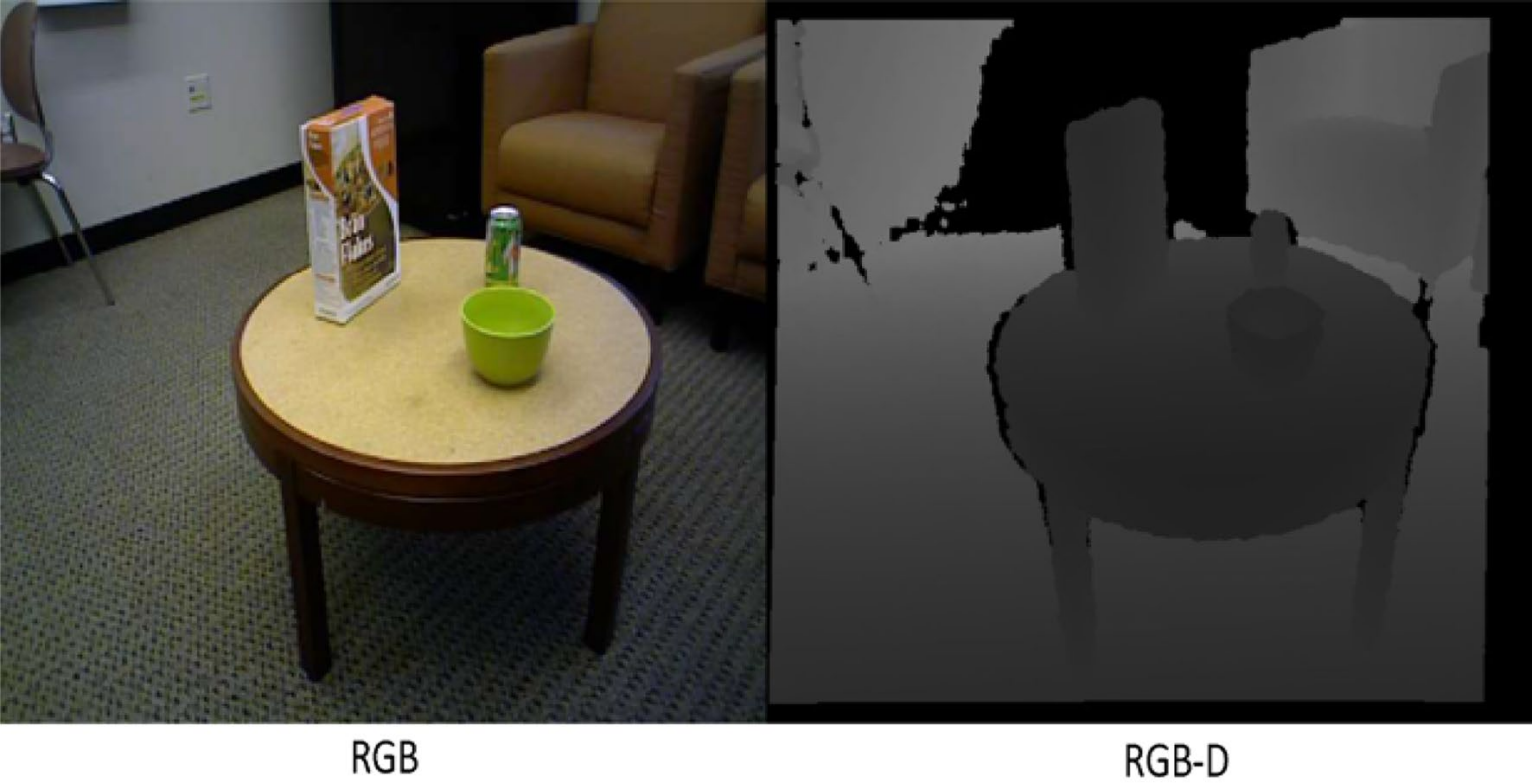
Depth based object dataset^19^.

Figure 5 demonstrates the utilization of photographs to communicate the distance of an object from the screen. Objects that are closer to the camera are depicted with greater pixel values, while those that are further away display lower pixel values. This visual representation depicts the depth information contained in the photos, presenting a gradient that correlates to the spatial organization of objects relative to the camera^16,17^. The fluctuation in pixel intensity functions as a visual cue for determining the relative distances between objects in a scene. This demonstrates the efficacy of using image-based depth representation to perceive spatial relationships in applications like depth sensing or 3D perception^18,19^.

**Figure 6:**
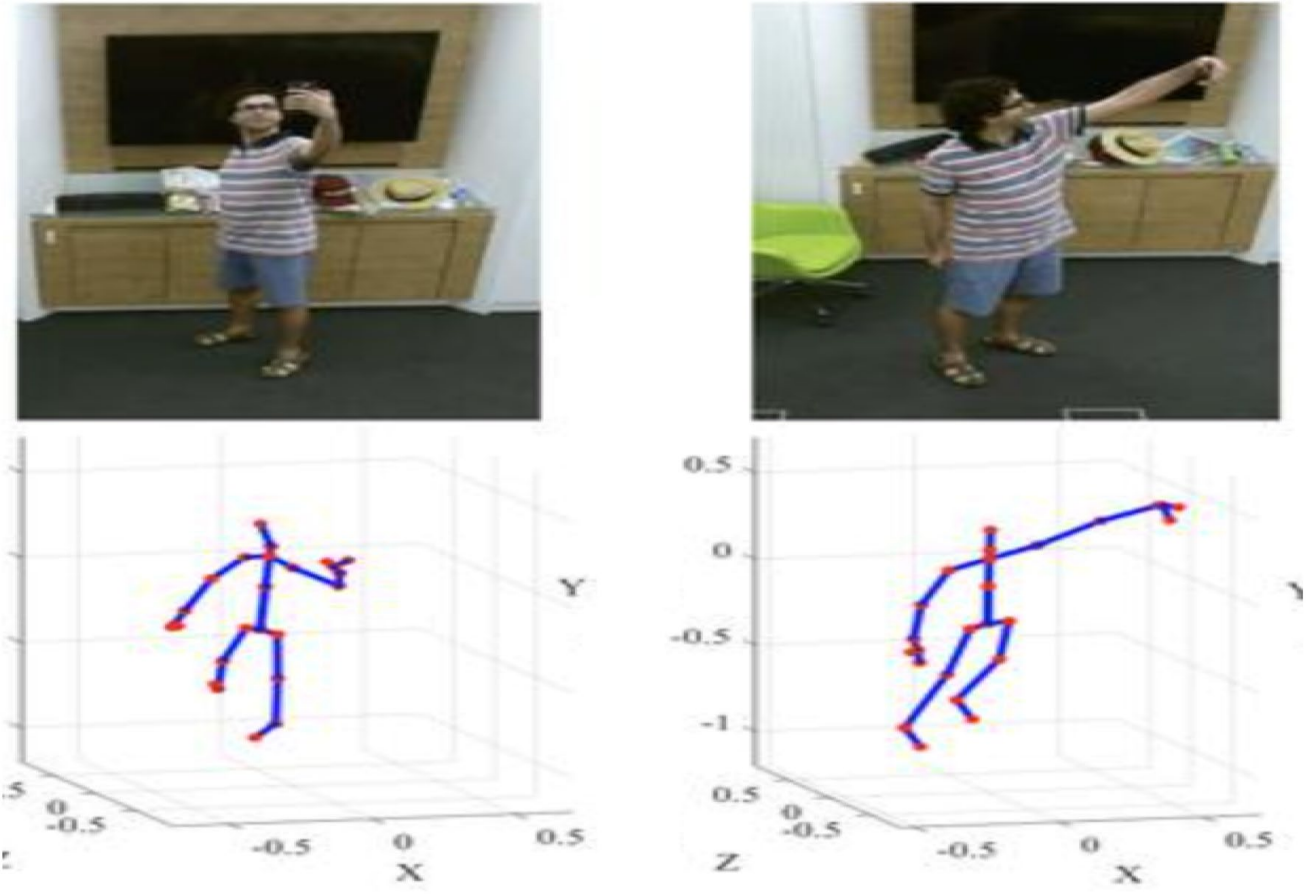
Skeleton based object dataset^20^.

Figure 6 displays a skeleton-based object dataset sourced from reference^20^. This dataset likely comprises skeletal representations of objects, where key joints and structural points are outlined to form a comprehensive dataset for training and evaluating models in tasks such as object recognition, pose estimation, or activity analysis. The use of skeleton-based datasets is common in computer vision and machine learning, providing a structured and informative representation of objects or human activities. The details and content of the dataset, as referenced in^20,21^, would provide additional insights into the specific characteristics and applications associated with this skeleton-based object dataset^22^. Table 1 provides a comprehensive overview of various feature-based state-of-the-art methods for action recognition. The methods employ different data types, datasets, and demonstrate performance metrics. Here is a summary of the representation methods bold in the table.

**Table 1:** Feature-based state-of-the-art methods for action recognition.

Method	Data type	Dataset	Performance	References
Fast Fourier transform	RGB	UCF101, Kinetics	Accuracy: 99.21	^11^
QSVM	RGB	UCF11, HMDB51	Accuracy: 94.43	^12^
SVM	RGB	UCSDped-1, UCSDped-2, UMN	Accuracy: 97.14	^13^
SVM	RGB	UCF11, UCF50	Accuracy: 78.6	^14^
SVM	RGB	MSRAction3D, UTKinectAction	Accuracy: 94.3	^15^
SVM	RGB	Weizmann, KTH, Hollywood2	**Accuracy: 86.3**	^16^
SVM	RGB	KTH, Weizmann, i3Dpost, Ballet, IXMAS	Accuracy: 95.5	^17^
SVM	RGB	KTH, UCFSports, Hollywood2	Accuracy: 91.8	^18^
SVM with ASAGA	RGB	UCSDped 1	Accuracy: 87.2	^19^
SVM with PSO	Skeleton	MSRAction3D, UT Kinect, Florence3D action	Accuracy: 93.75	^20^
SVM with GA	RGB	KTH, HMDB51, UCF YouTube, Hollywood2	Accuracy: 95.0	^21^
SVM-neural network	RGB	KTH, Weizmann	Average Accuracy: 96.4	^22^
RF	Skeleton	UT Kinect	Accuracy: 92	^23^
NBNN	3D joints skeleton	MSRAction3D-Test1, MSRAction3D-Test2, MSRAction3D-cross-subject	Accuracy: 95.8	^24^
HMM-Kernel Discriminant analysis	Silhouette	Elder care data	Accuracy: 95.8	^25^
HMM	Skeleton	Im-DailyDepthActivity, MSRAction3D (CS), MSRDailyActivity3D (CS)	Accuracy: 74.23	^26^

### Convolutional neural network based action recognition

Convolutional Neural Networks (CNNs) have emerged as a powerful and effective approach for action recognition in video data. Leveraging their ability to automatically learn hierarchical features from visual data, CNNs have significantly contributed to the advancements in human action recognition. the key aspects of CNN-based action recognition are Spatial–Temporal Hierarchical Features, 3D Convolutional Networks (3D CNNs), Two-Stream CNNs, Pre-trained Models, Long-Short Term Memory Networks (LSTMs) Integration, Attention Mechanisms, Real-Time Action Recognition, Diverse Datasets, The continual evolution of CNN-based models, coupled with innovations in architecture design and training strategies, positions them as a cornerstone in the field of human action recognition, providing state-of-the-art performance in diverse and dynamic video datasets^23^. This Table 2 provides an overview of various Convolutional Neural Network (CNN) based methods for action recognition, encompassing diverse datasets and performance metrics. The methods include PoseConv3D leveraging RGB + Depth data from NTU-RGBD, Temporal Difference Networks applied to Something-SomethingV1 and Kinetics datasets, CNN models trained on UCF101, HMDB51, FCVID, and Activity Net, 2-stream Convolution Network on UCF101 and HMDB51, 3-stream CNN on KTH, UCF101, and HMDB51, Multi-stream CNN utilizing Skeleton data from NTU-RGBD, MSRC-12, and Northwestern-UCLA, 3D CNN on KTH, from NTU-RGBD and Kinetics, and additional CNN models applied to various datasets such as UCF101, HMDB51, UCF50, action, and HMDB51 with unique performance metrices^24^.

**Table 2:** Convolutional neural network based action recognition.

Method	Data type	Dataset	Performance	References
PoseConv3D	RGB + Depth	NTU-RGBD	Accuracy: 69.4, 94.2	^1^
Temporal difference networks	RGB	Something-SomethingV1, Kinetics	Accuracy: 68.2, 79.4	^2^
CNN	RGB	UCF101, HMDB51, FCVID, Activity Net	Accuracy: 98.6, 84.3, 82.1, 84.4	^3^
2-Stream convolution network	RGB	UCF101, HMDB51	Accuracy: 91.5, 65.9	^4^
3-Stream CNN	RGB	KTH, UCF101, HMDB51	Accuracy: 96.8, 92.2, 65.2	^5^
Multi-stream CNN	Skeleton	NTU-RGBD (CS), NTU-RGBD (CV), MSRC-12 (CS), Northwestern-UCLA	Accuracy: 80.03, 87.21, 96.62, 92.61	^6^
3D CNN	RGB	UCF101, HMDB51	Accuracy: 90.2	^7^
Actional-graph-based CNN	Skeleton	UCF50, UCF101, YouTube action, HMDB51	Accuracy: 86.8, 94.2, Top-5 acc: 56.5, Top-1 acc: 34.8	^8^
CNN	RGB	UCF50	Accuracy: 92.5, 65.2	^9^
CNN	RGB	UTD-MHAD, NTU-RGBD (CV), NTU-RGBD (CS)	Accuracy: 96.4, 94.33, 96.21, 70.33	^10^
CNN-genetic algorithm	RGB	UCF50	Accuracy: 99.98	^11^
CNN	Skeleton	UTD-MHAD, NTU-RGBD (CV), NTU-RGBD (CS)	Accuracy: 88.10, 82.3, 76.2	^12^

### RNN based action recognition

In the realm of action recognition, Recurrent Neural Networks (RNNs) have garnered considerable attention for their capacity to model sequential dependencies within temporal data, making them particularly apt for video analysis. Unlike traditional feedforward networks, RNNs possess a memory mechanism that enables them to capture and retain information over time, crucial for understanding dynamic actions. Key aspects of RNN-based action recognition. Are Temporal Modeling, Long Short-Term Memory (LSTM) Networks, Bi-directional RNNs, Spatial–Temporal Interaction Modeling, Skeleton-based Action Recognition.

**Figure 7:**
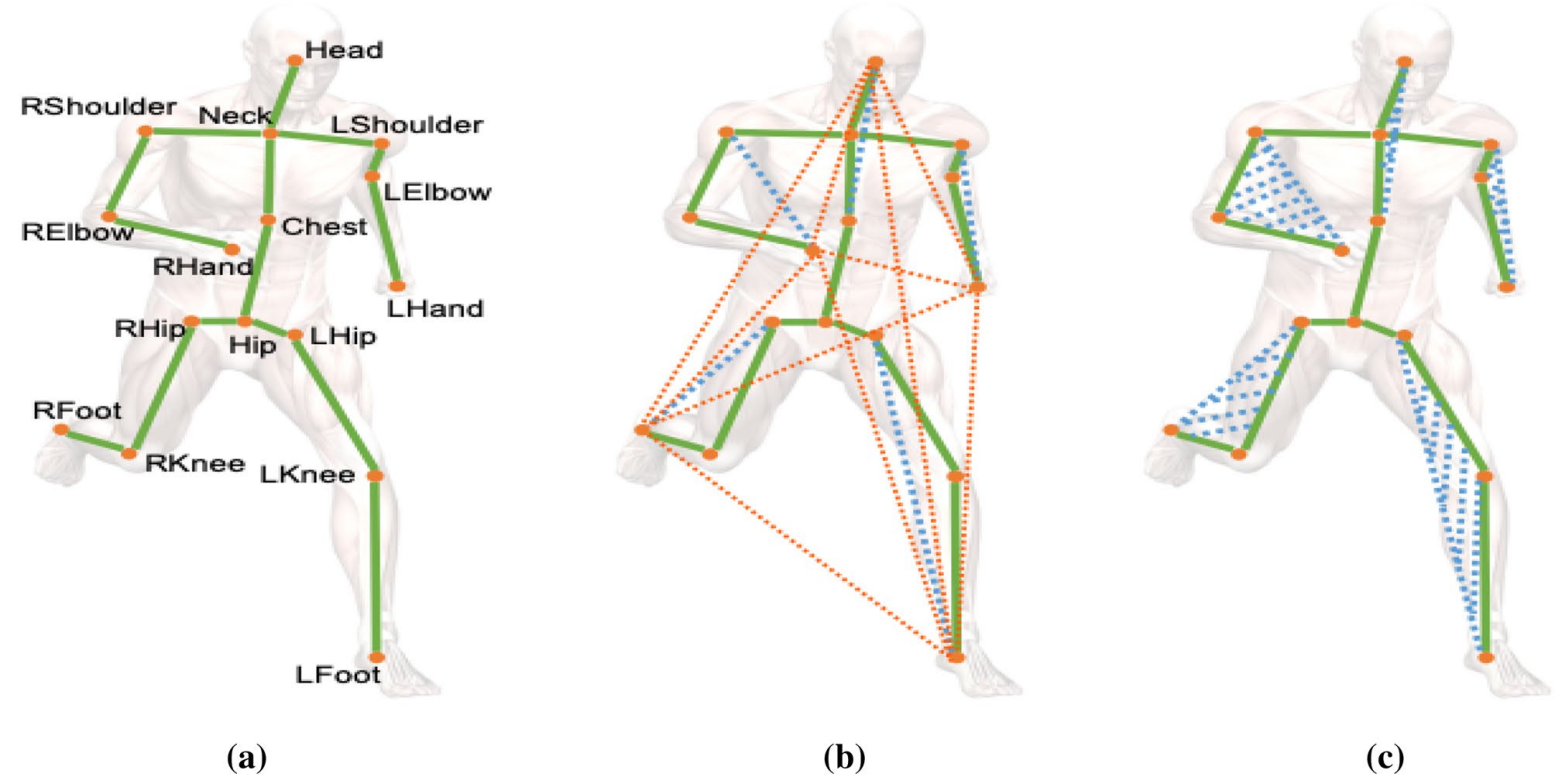
Evolution of RNN based action recognition^25^.

**Figure 8:**
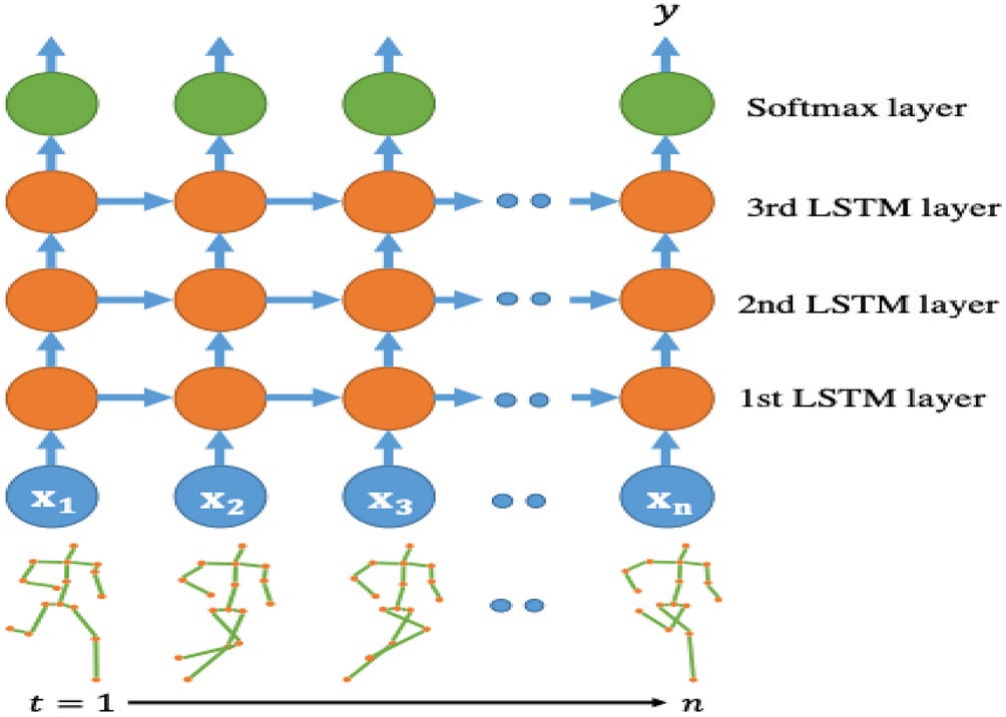
RNN based—LSTM network^25^.

Figure 7 depicts the progression of action recognition approaches based on RNN over time. The progression of RNN structures, encompassing the integration of sophisticated variations such Long Short-Term Memory (LSTM) networks, bidirectional RNNs, and attention mechanisms. This Fig. 8 likely illustrates the architecture and components of an RNN-based model specifically leveraging Long Short-Term Memory (LSTM) networks for action recognition.

### Activities based action recognition

Activities-based Action Recognition typically refers to the recognition and classification of human actions based on various activities performed in a given context.

1. Body Motion:

Movements such as walking, running, jumping, or other fundamental body motions fall under this category.

2. Human–Human Interactions:

Actions involving communication, gestures, conversations, or any form of interaction between individuals.

3. Human–Object Interactions:

Actions related to interactions with physical objects, such as picking up an item, manipulating tools, or any engagement with the environment.

4. Playing Musical Instruments:

Actions involving the performance of playing musical instruments, showcasing a skill or artistic expression.

5. Sports:

Physical activities associated with sports, including running, jumping, throwing, and other dynamic movements within a sports context.

## Exploration of well-known public datasets and methods for human action recognition

**Figure 9:**
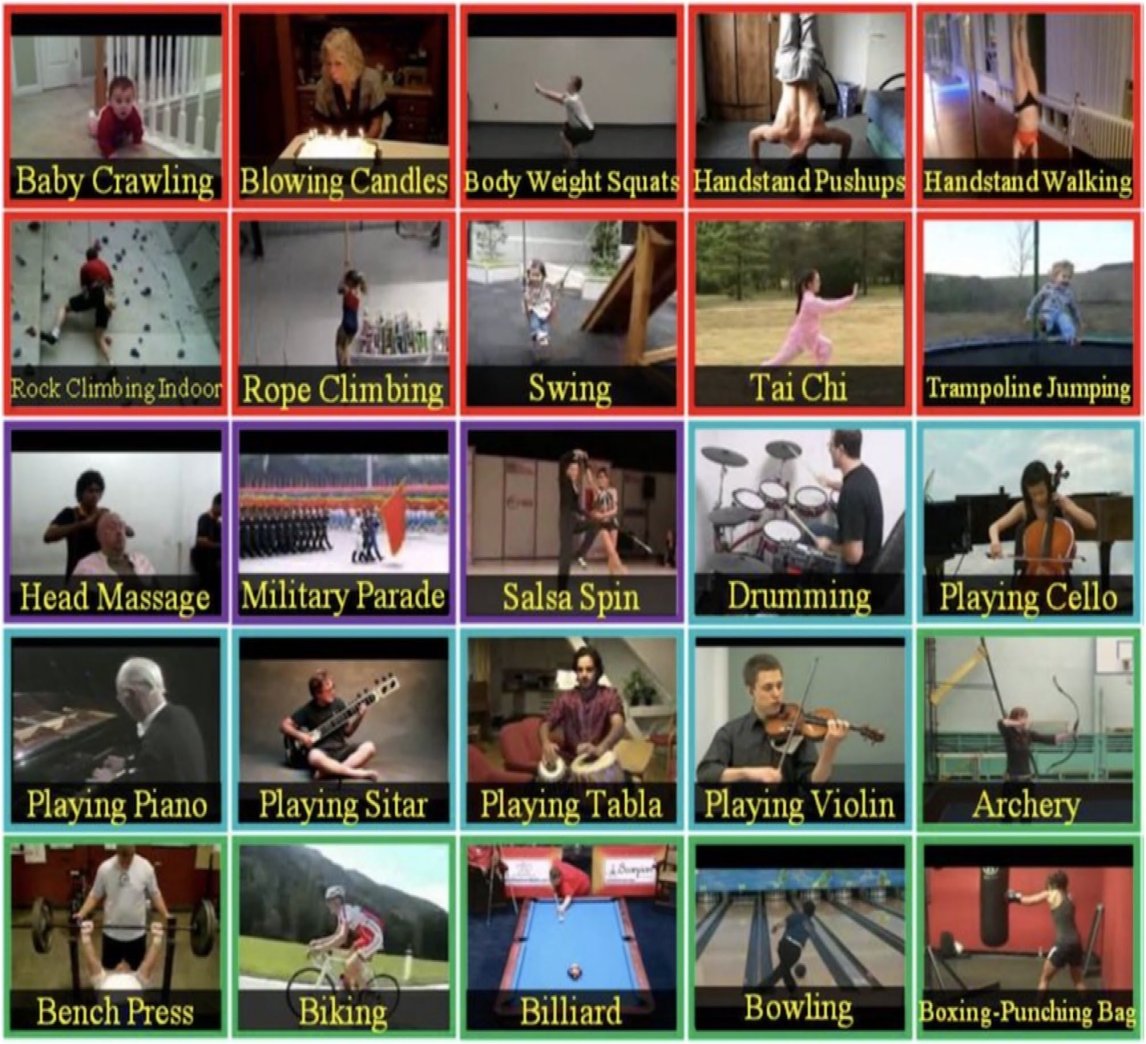
Activity based action recognition UCF101 human actions dataset^26^.

Figure 9 Activity based Action Recognition UCF101 Human Actions dataset^26^ from the link (https://github.com/OpenGVLab/VideoMAEv2). The exploration of well-known public datasets and methods for human action recognition is an essential aspect of understanding the current state of the field and evaluating the performance of different algorithms^26^. This work has derived datasets from UCF101 Human Actions dataset. This dataset is made up of 101 different action categories that represent a wide range of real-life everyday tasks. The dataset shows the complexity of real-life situations by including a lot of different activities, such as sports, everyday life, and relationships that are hard to understand. Researchers use UCF101 to test and improve computer vision techniques, which helps the ongoing development of recognizing human behaviour. Human actions are diverse and encompass a wide range of activities. Here are different types of human actions categorized based on various contexts: Everyday Activities: Walking, Running, Sitting, Standing, Eating, Drinking.

Gestures and Expressions: Waving, Pointing, Nodding, Clapping, Thumbs-up, Facial expressions.

Sports and Physical Exercises: Jumping, Dancing, Swimming, Playing basketball, soccer, tennis, etc., Yoga poses, Weightlifting.

Work-related Activities: Typing, Writing, using tools, operating machinery, Presenting.

Interactions: Handshakes, Hugging, High-fives, Conversations, Collaborative activities.

Recreational Activities: Playing musical instruments, Painting, Reading, playing video games, Watching TV/movies.

Health and Wellness: Exercising, Stretching, Meditating, Running/jogging, Cycling.

Emergency Actions: Evacuating, Running for safety, First aid gestures, Alert expressions.

Transportation-related Actions: Driving, Cycling, boarding a vehicle, Walking on a busy street.

Educational Activities: Attending lectures, Studying, taking notes, Participating in experiments.

### Public datasets

1. HMDB51 (Human Motion Database):

It’s a popular dataset with a focus on actions in realistic settings, consisting of 51 action categories.

2. Kinetics:

A large-scale dataset with a broad range of human actions, comprising videos sourced from YouTube, providing a significant challenge for action recognition models.

3. NTU RGB + D:

This dataset includes RGB and depth information, capturing human actions in various scenarios, making it suitable for both 2D and 3D action recognition.

4. Something-SomethingV1:

Datasets designed for fine-grained action recognition, involving actions related to manipulating everyday objects.

**Figure 10:**
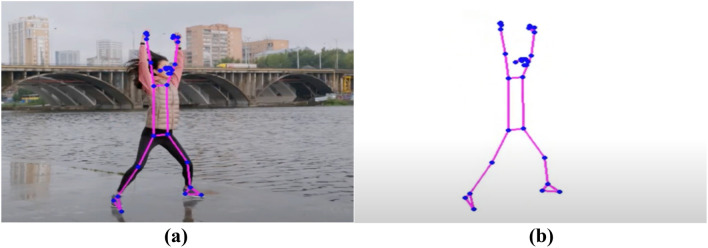
HMDB51 dataset sample^27^.

**Figure 11:**
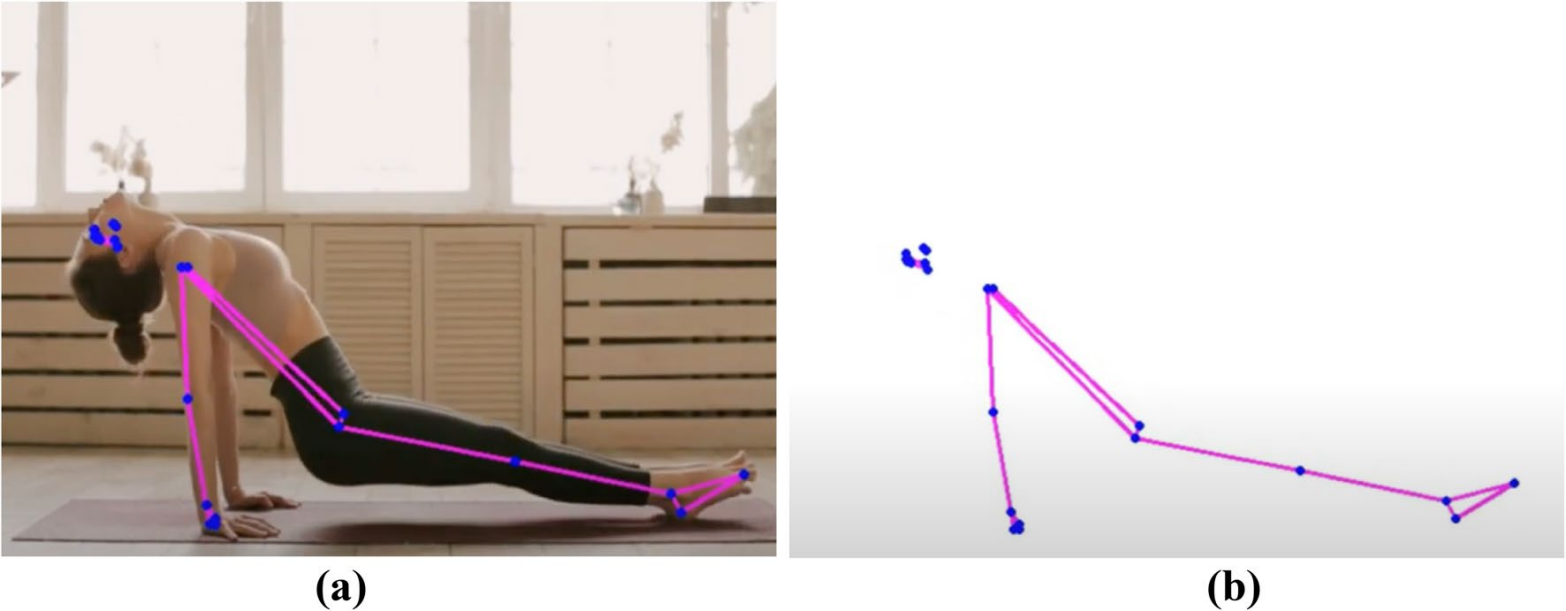
Kinetics dataset sample^27^.

**Figure 12:**
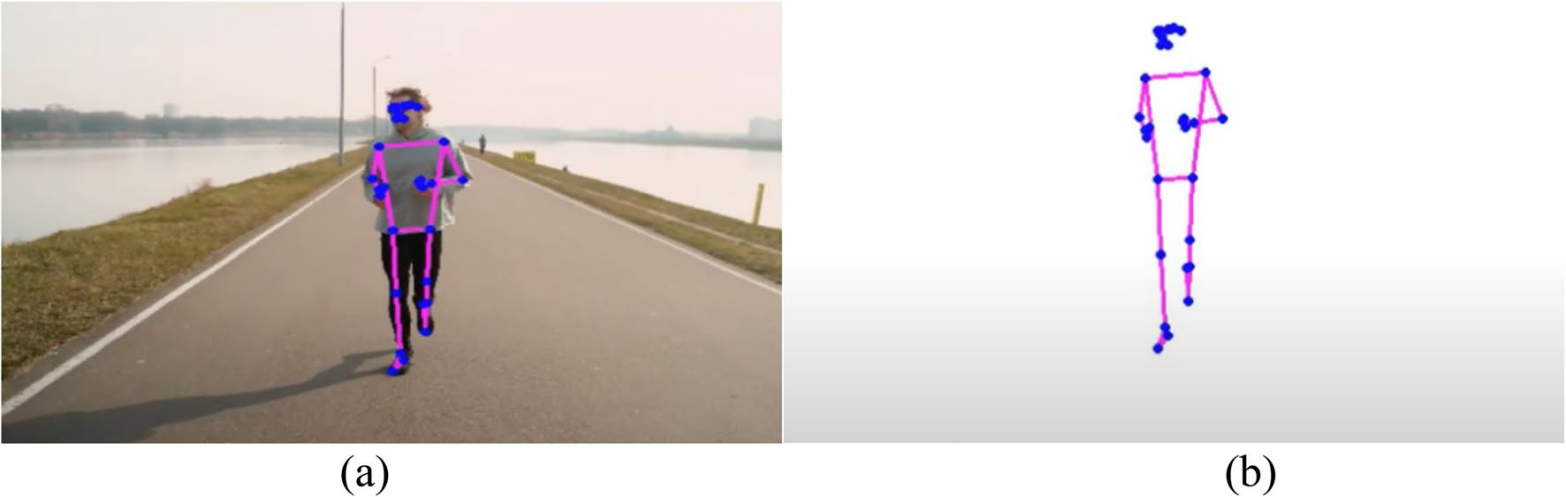
NTU RGB + D dataset sample^27^.

**Figure 13:**
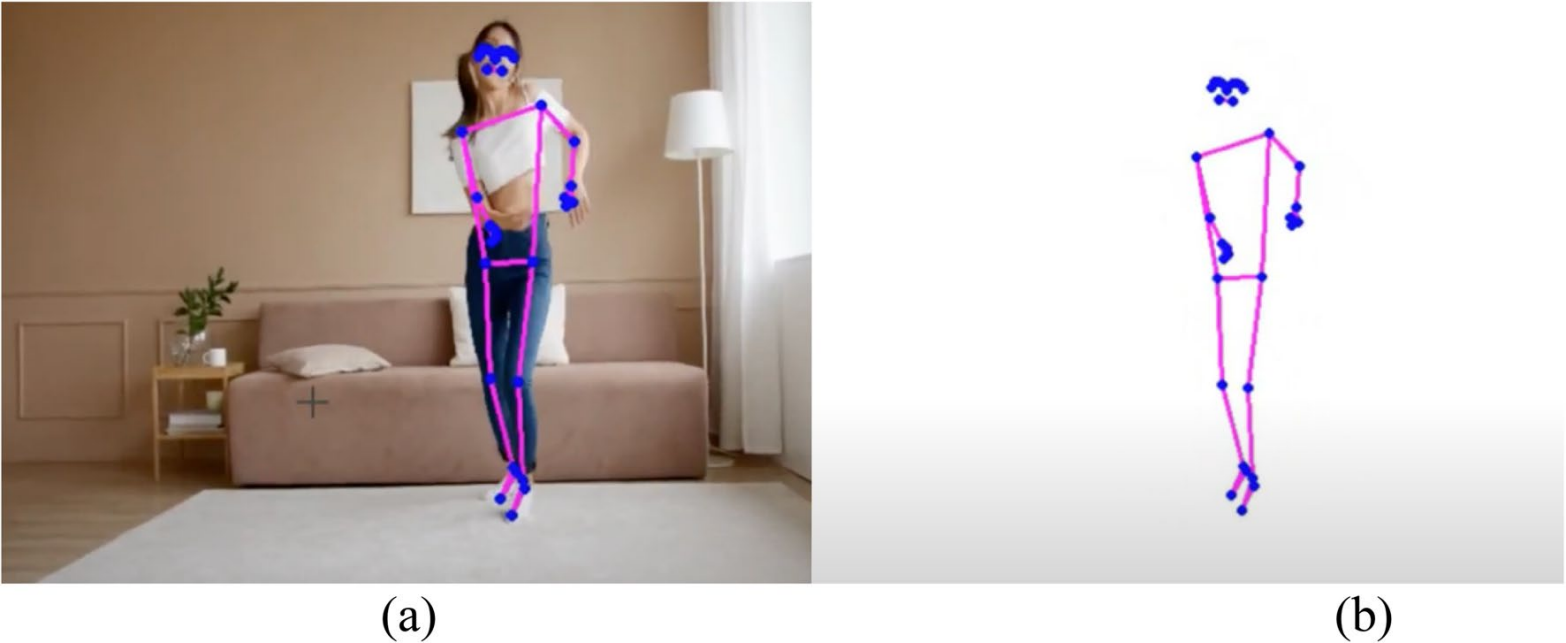
Something SomethingV1Dataset sample^27^.

Figure 10 showcases samples from the HMDB51 dataset, Fig. 11 illustrates examples from the Kinetics dataset, Fig. 12 exhibits samples from the NTU RGB + D dataset, and Fig. 13 presents instances from the Something SomethingV1 dataset. These visual representations are crucial for understanding the diverse range of actions and scenarios captured in each respective dataset^27^

## Evaluation of metrics and performance

Evaluation metrics commonly used in human action recognition is Accuracy, Precision, Recall, F1 Score. And the confusion matrix is explained with the below mentioned example, Let’s say that you are having trouble classifying human actions, and your confusion matrix looks like this:

$$ \left[ {\begin{array}{*{20}c} {500} & {20} \\ {10} & {470} \\ \end{array} } \right] $$

This implies:

TP (True Positives): 470.

TN (True Negatives): 500.

FP (False Positives): 20.

FN (False Negatives): 10.

So the interpretation would be,

1 $$ {\text{Accuracy}} = \frac{470 + 500}{{470 + 500 + 10 + 20}} \approx \, 0.{97}\;{\text{or}}\;{97}\% $$

2 $$ {\text{Precision}} = \frac{470}{{470 + 20}} \approx \, 0.{95}\;{\text{or}}\;{95}\% $$

3 $$ {\text{Recall}} = \frac{470}{{470 + 10}}{ } \approx \, 0.{979}\;{\text{or}}\;{97}.{9}\% $$

4 $$ {\text{F}}1{\text{ Score}} = 2{ } \times { }\frac{{0.95{\text{x}}0.979}}{0.95 + 0.979} \approx 0.{969}\;{\text{or}}\;{96}.{9}\% $$

## Challenges, prospects, and forthcoming trends in human activity recognition

### Challenges in the field of human activity recognition

1. It is difficult to create models that work well in a variety of circumstances because human behaviours can vary greatly in terms of pace, style, and execution^28^.
2. The accuracy of human activity detection systems can be greatly affected **by factors** such as illumination, occlusion, and background clutter.
3. Feature fusion and model creation are complicated when integrating data from many modalities, like video, depth, and sensor data, to improve recognition accuracy^29^.
4. Creating models that effectively handle growing data volumes while preserving real-time performance is a never-ending task, particularly for large-scale applications^30^.
5. The continual difficulty lies in developing models that can efficiently handle the increasing volume of data and maintain real-time performance, particularly for large-scale applications.

### Prospects in the field of human activity recognition

1. Ongoing progress in deep learning methods, including innovative structures and pre-training approaches, have the capacity to enhance the precision and resilience of models for recognising human activities^31^.
2. Integration with edge computing and the Internet of Things (IoT) has the potential to facilitate real-time processing and decision-making, resulting in decreased latency and improved practical use of recognition systems.
3. Interpretability in AI refers to the ability to understand and explain the reasoning behind the decisions made by an AI system.

### Forthcoming trends in human activity recognition

1. Self-supervised learning methods, which involve models acquiring knowledge from unlabelled data, are becoming increasingly popular for the purpose of human activity recognition^32^. This technique helps to decrease the dependence on labelled datasets.
2. The inclination towards constructing more comprehensible models corresponds to the increasing need for responsibility and openness in AI systems, particularly in applications that have an impact on human lives.
3. The utilisation of attention processes and transformer topologies in models for human activity recognition is projected to increase^33^. This will enable models to concentrate on pertinent spatial and temporal characteristics.
4. There is a growing focus on multimodal learning, which involves integrating data from several sensors and modalities, in order to create more complete and precise representations of human behaviours.
5. There is a movement towards implementing and incorporating human activity recognition into real-world settings, particularly in industries like healthcare, security, and smart environments^34^. This trend focuses on practical applications and the significant effects it can have.
6. Gaining insight into these obstacles, opportunities, and upcoming patterns is essential for designing the future of human activity recognition and meeting the changing requirements of different industries and applications^35^.

## Conclusion

In conclusion, this systematic survey delves into the landscape of Human Activity Recognition (HAR) with a specific focus on HARNet, a Deep Learning-based approach^36,37^. The exploration encompasses a comprehensive review of the existing literature, providing insights into the evolution, challenges, and advancements in HAR methodologies. HARNet, as a notable player in the field, is scrutinized for its contributions and efficacy in addressing the intricacies of recognizing human actions. Through a systematic and structured analysis, this survey contributes to the understanding of HAR methodologies, offering a valuable resource for researchers, practitioners, and enthusiasts in the domain. As technology continues to evolve, HARNet and its counterparts stand as integral components in harnessing the potential of Deep Learning for accurate and robust human activity recognition, paving the way for future innovations and applications in diverse real-world scenarios.

## Data Availability

The data used to support the findings of this study are included in the article.
